# Tranexamic versus aminocaproic acids in patients with total hip arthroplasty: a retrospective study

**DOI:** 10.1186/s12891-022-05922-5

**Published:** 2022-11-19

**Authors:** Hui Xie, Yu-Shen Yang, Si-miao Tian, Ben-jie Wang, Wei-min Fu, Liang-liang Cheng, Nan-nan Jiang, Guishan Gu, De-wei Zhao

**Affiliations:** 1 Department of Orthopaedic, Affiliated Zhongshan Hospital of Dalian University, Dalian, 116001 Liaoning Province People’s Republic of China; 2 Dalian University Affiliated Xinhua Hospital, Dalian, 116000 Liaoning Province People’s Republic of China; 3 Department of Orthopaedic, The First Hospital of Jilin University, Changchun, 130021 Jilin Province People’s Republic of China

**Keywords:** Total hip arthroplasty, Transfusion, Tranexamic acid, Epsilon aminocaproic acid, Cost savings

## Abstract

**Background:**

Recently, tranexamic acid (TXA) and epsilon aminocaproic acid (EACA) have been applied in total hip arthroplasty (THA). However, doubts in clinicians’ minds about which medicine is more efficient and economical in THA need to be clarified. Therefore, this study compared the efficacy and cost of the intraoperative administration of TXA and EACA per surgery in decreasing perioperative blood transfusion rates in THA.

**Methods:**

This study enrolled patients who underwent THA between January 2019 to December 2020. A total of 295 patients were retrospectively divided to receive topical combined with intravenous TXA (*n* = 94), EACA (*n* = 97) or control (*n* = 104). The primary endpoints included transfusions, estimated perioperative blood loss, cost per patient and the drop in the haemoglobin and haematocrit levels.

**Results:**

Patients who received EACA had greater total blood loss, blood transfusion rates, changes in HGB levels and mean cost of blood transfusion per patient (*P* < 0.05) compared with patients who received TXA. In addition, both TXA and EACA groups had significantly fewer perioperative blood loss, blood transfusion, operation time and changes in haemoglobin and haematocrit levels than the control group (*P* < 0.05). Cost savings in the TXA and EACA groups were 736.00 RMB and 408.00 RMB per patient, respectively.

**Conclusions:**

The application of perioperative antifibrinolytics notably reduces the need for perioperative blood transfusions. What’s more, this study demonstrated that TXA is superior to EACA for decreasing blood loss and transfusion rates while at a lower cost per surgery. These results indicate that TXA may be the optimum antifibrinolytics for THA in Chinese area rather than EACA.

**Supplementary Information:**

The online version contains supplementary material available at 10.1186/s12891-022-05922-5.

## Introduction

Total hip arthroplasty (THA) is a successful surgical procedure for the treatment of various end-stage hip diseases, such as femoral head necrosis, osteoarthritis and femoral neck fracture. A report and analysis of amount of hip and knee arthroplasty in China from 2011 to 2019 revealed that the annual amount of THA in China in 2019 was 577,153 with annual growth rate of 16.67% and there is an increase in demand each year [1]. However, this process is associated with substantial perioperative blood loss, which greatly increases the transfusion requirements [2]. Blood transfusions are linked to many adverse events, consisting of haemolytic transfusion reactions, postoperative infections and longer length of hospital stay [3]. Besides, the high cost of blood- and transfusion-related activities imposes additional financial burdens on many patients undergoing THA and receiving allogeneic red blood cell (RBC) transfusions. Therefore, it is urgent to find an optimal blood-loss minimizing strategy to reduce operative costs and improve patient outcomes.

Previously, multiple blood conservation practices, including spinal anaesthesia, red blood cell salvage suction tips, tourniquets, and reinfusion drains, have been applied in the clinic, but all of these practices have had varying limitations [4]. Fortunately, the use of antifibrinolytics has been demonstrated to be a more effective measure for perioperative blood management than former strategies. Tranexamic acid (TXA) and epsilon aminocaproic acid (EACA) are the most common antifibrinolytic medications, with basically similar antifibrinolytic mechanisms [5]. Previous studies have shown that both antifibrinolytics are correlated with an obvious decrease in perioperative blood loss and the need for blood transfusions in orthopaedic [6] surgery. Recently, some reports indicated that EACA and TXA have similar efficacy in THA for reducing blood loss and transfusion requirements, while EACA seems to be more economic than TXA [7]. Therefore, EACA was recommended to replace TXA in orthopedic surgery.

In fact, not only Western countries, but many Chinese medical institutions already do this. However, no direct clinical evidence showed the cost advantage of EACA over TXA to be seen in Chinese areas so far. On the contrary, at our institution, drug price for TXA and EACA were 51.38 RMB per 1 g and 117.86 RMB per 4 g, respectively, which as it literally shows, means EACA costs almost twice as much as TXA in China. Obviously, Western guide is inapplicable to Chinese actual situation. With the rapid growth of annual amount of THA in China, finding the optimum antifibrinolytics in Chinese area based on cost and regional availability will offer significant medical cost savings. Therefore, this study was designed to compare the efficacy and cost of intraoperative administration of TXA and EACA in THA and to preliminarily provide clinical evidence for the choice of antifibrinolytics in Chinese areas.

## Material and methods

A total of 295 patients who underwent primary THA in our hospital from January 2019 to December 2020 were retrospectively divided to receive topical combined with intravenous TXA (*n* = 94), EACA (*n* = 97) or control (*n* = 104, normal saline). Antifibrinolytics were given twice perioperatively. Tranexamic Acid and Sodium Chloride Injection (1 g EACA/100 ml normal saline, 51.38 RMB per bottle) and Aminocaproic Acid and Sodium Chloride Injection (4 g EACA/100 ml normal saline, 117.86 RMB per bottle) were purchased from Chengdu Bette Pharmaceutical Co., LTD. The first bottle of TXA and EACA was given intravenously before making the incision, and a second was given topically during wound closure. The study design was approved by the Ethics Committee of Affiliated Zhongshan Hospital of Dalian University (Approval number, 2021045). Patients who had been preoperatively administered anticoagulants and antiplatelet drugs and those with chronic heart failure or ischaemic heart disease, chronic hepatic or renal failure, thromboembolic episodes, a history of hip surgery, idiopathic osteonecrosis of the femoral head, rheumatoid arthritis, or a preoperative haemoglobin (HGB) level below 8 g/dL were excluded. All surgeons utilized the same surgical instruments and standardized operative procedures. All THA processes were performed under general anaesthesia using a lateral approach [8]. Cementless stems and cups were used in all cases. To allow adequate drainage, hemovac drains were routinely placed at the wound site under fascias during closure. Then, hemovac drains were removed when the drainage volume was less than 40 mL per 8-h shift. The study was conducted in accordance with the Basic & Clinical Pharmacology & Toxicology policy for experimental and clinical studies [9].

All patients followed the same clinical pathway, including standard postoperative care, analgesia, and antithrombotic therapy. Indications for blood transfusion were as follows: (i) HGB of less than 7 g/dl in patients without cardiovascular disease [10]; (ii) HGB of 8 to 9 g/dl in patients accompanied by established cardiovascular risk factors or disease; (iii) HGB below 10 g/dl in patients with poor clinical tolerance of lower values; (iv) symptoms of anaemia, including vertigo, hypotension, and bradycardia; and (v) symptoms of hypoxia, such as tachycardia, dyspnoea, or syncope [11].

Intraoperative blood loss, changes in HGB and haematocrit (HCT) from preoperative levels to the first postoperative day, and transfusion rates were recorded. Intraoperative blood loss was determined based on the contents of the suction bottle and the change in the weight of the surgical sponges used. Postoperative drainage was calculated using the volume of blood in hemovac drains. The respective sum of the intraoperative blood loss and postoperative drainage was the total loss for that patient.

The primary endpoints included transfusions, estimated perioperative blood loss, cost per patient, and drops in HGB and HCT levels. The secondary indexes consisted of operation time, postoperative complications, and length of hospital stay.

### Statistical analysis

SPSS version 20.0 software (IBM Corp., Armonk, NY, USA) was used for the statistical analyses. All results from measurement data in the present study are expressed as the mean ± SD. Comparisons of the three groups were performed using one-way analysis of variance (ANOVA) tests, followed by Tukey's test for post-hoc comparisons, or the χ^2^ test for categorical variables. P-values less than 0.05 were considered significant.

## Results

Of the 295 primary THA cases identified for inclusion during the 24-month study period, 94 patients received TXA, 97 patients received EACA, and 104 patients received no antifibrinolytics. The patient characteristics are given in Table 1. No statistically significant differences were noted in the age, sex proportion, height, weight, body mass index, disease constitution, or hospitalization time among the groups (*P* > 0.05).

**Table 1:** Demographic characteristics of 295 patients who underwent total hip arthroplasty

**Demographics**	**Control** **(*n* = 104)**	**EACA** **(*n* = 97)**	**TXA** **(*n* = 94)**
Mean (and SD) age, yr	61.80 (9.641)	63.31 (8.237)	63.89 (8.430)
Gender, male: female	49: 55	36: 61	29: 65
Diagnosis, no. (and %) of patients			
Toxic osteonecrosis	29 (27.9)	25 (25.8)	30 (31.9)
Dysplastic hips	35 (33.7)	24 (24.7)	22 (23.4)
Femoral neck fractures	22 (21.2)	29 (29.9)	31 (33.0)
Hip osteoarthritis	14 (13.5)	18 (18.6)	9 (9.6)
Other hip diseases	4 (3.8)	1 (1.0)	2 (2.1)
Mean (and SD) body mass index, kg/m^2^	24.73 (3.263)	24.19 (3.088)	23.83 (3.628)
Mean (and SD) operation time, min	89.45 (39.975)	74.80 (29.795)^*^	70.00 (17.629)^†^
Mean (and SD) hospitalization time, day	12.02 (3.478)	11.51 (4.435)	11.23 (3.697)
Mean (and SD) postoperative hospitalization time, day	8.19 (1.199)	7.96 (1.190)	8.00 (1.117)

*TXA* tranexamic acid, *EACA* epsilon aminocaproic acid

^*^P < 0.05 for the comparison between EACA and control group

^†^*P* < 0.05 for the comparison between TXA and control group

### Comparison of the TXA or EACA group to control group

**Fig. 1:**
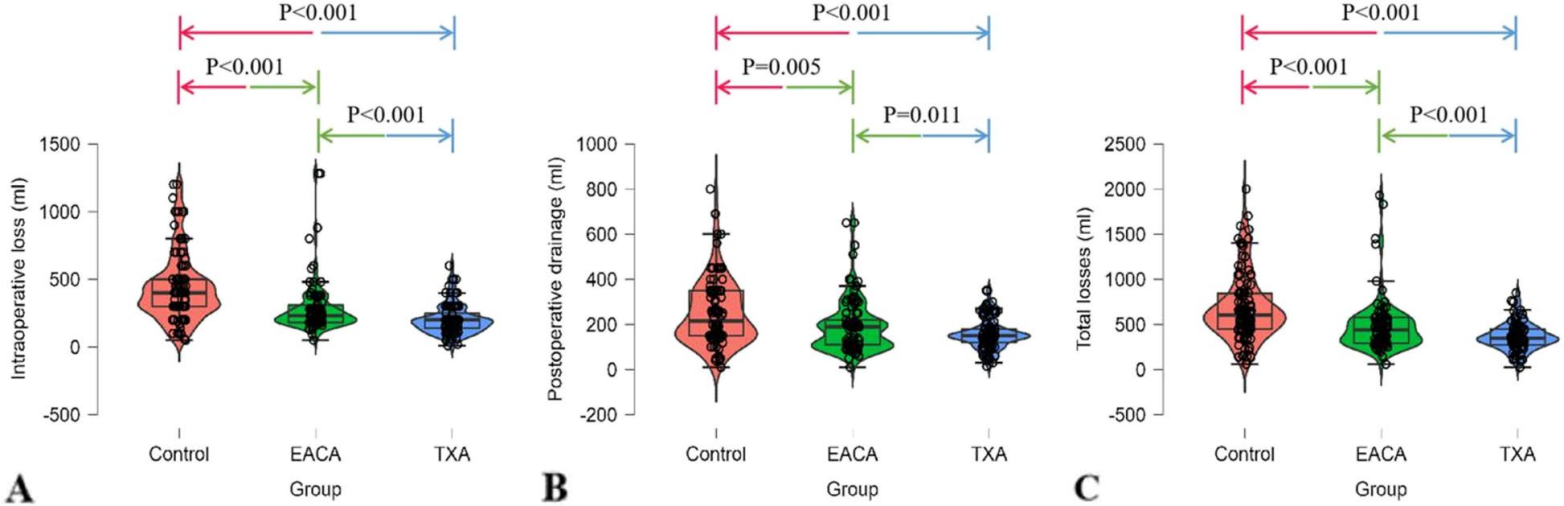
Comparison of perioperative blood loss among three groups. **A** Intraoperative blood loss. **B** Postoperative blood loss. **C** Total blood loss. Statistical significance was reached for total losses among tranexamic acid (TXA), epsilon aminocaproic acid (EACA), and control. Pairwise comparisons among the three groups were determined by Tukey's test

**Fig. 2:**
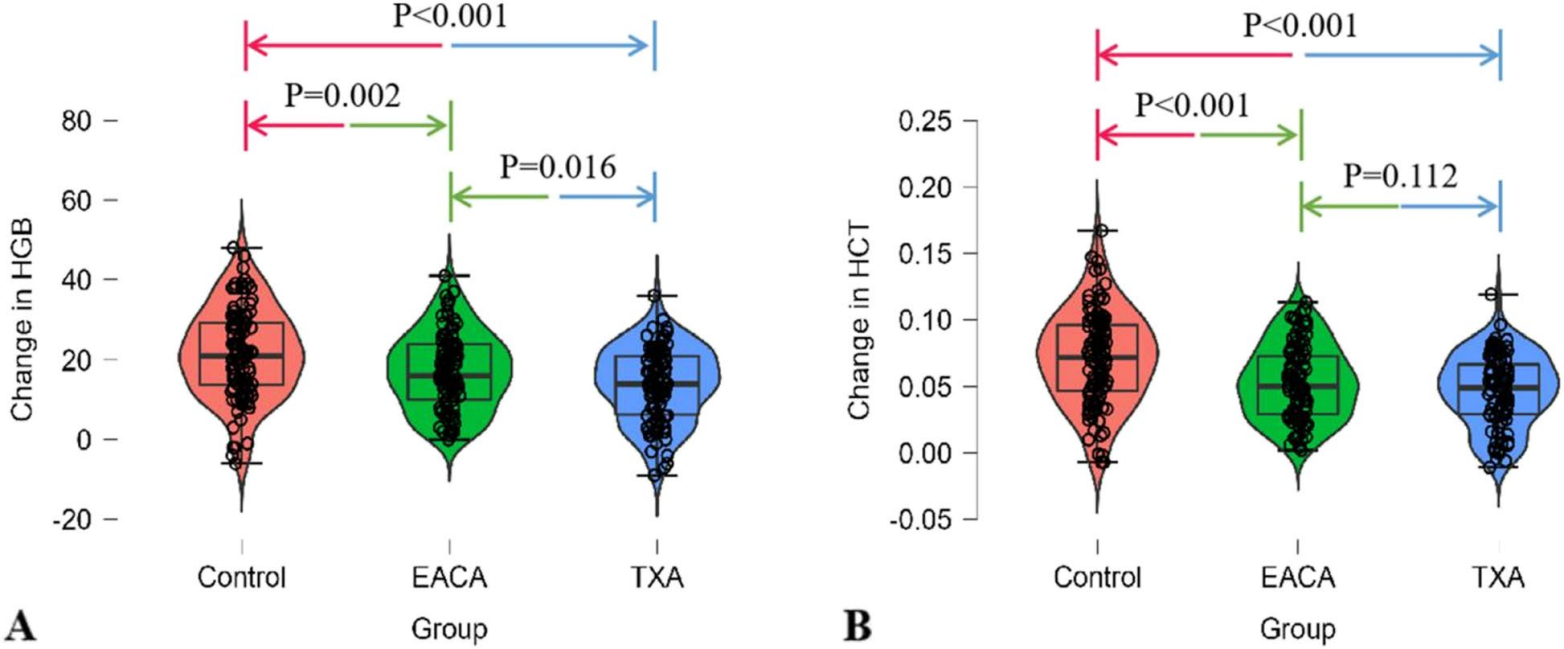
Change in haemoglobin (HGB) and haematocrit (HCT) levels in patients among the three groups. **A** Change of HGB level; **B** Change of HCT level. TXA, tranexamic acid; EACA, epsilon aminocaproic acid. Pairwise comparisons among the three groups were determined by Tukey's test

In the current study, the transfusion time mainly focused on the intraoperation, and 1st postoperative day based on haemoglobin level. The control group exhibited a notably higher transfusion rate than both the TXA and EACA groups, 36.5% compared with 10.6% and 20.6%, respectively (*P* < 0.05), as shown in Table 2. Similarly, the average amount of blood transfusion per patient was similar for TXA and EACA (0.21 and 0.62 units, respectively), and both were significantly lower than the 1.13 units for the control group (*P* < 0.05) (Table 2). The perioperative blood loss in the TXA and EACA groups, including intraoperative blood loss and postoperative drainage, was significantly lower than that in the control group (*P* < 0.001) (Fig. 1.). In addition, there were significantly greater decreases in HGB and HCT levels in patients who had been given control than in those who had received TXA (*P* < 0.001) or EACA (*P* < 0.005) (Fig. 2.). Furthermore, both the TXA and EACA groups presented shorter operation times than the control group, 70.00 min and 74.80 min vs. 89.45 min, respectively (*P* < 0.05) (Table 1).

**Table 2:** Transfusion financial summary

Indicators	Control	EACA	TXA
Perioperative transfusion rate	36.5% (38/104)	20.6% (20/97)^*^	10.6% (10/94)^†,§^
Mean perioperative transfusion no. of units	1.13	0.62^*^	0.21^†,§^
Mean cost of blood transfusion per patient (RMB)	900.00	498.97^*^	170.21^†,§^
Mean cost saving for transfusions (RMB) compared with control	-	408.00	736.00
Generalized cost saving for transfusions (RMB)	-	172.28	633.24

Transfusion cost assumes 800 RMB per unit red blood cell; TXA cost assumes 102.76RMB per 2 g; EACA cost assumes 235.72 RMB per 8 g

*TXA* tranexamic acid, *EACA* epsilon aminocaproic acid, *RMB* Renminbi

^*^P < 0.05 for the comparison between EACA and control group

†*P* < 0.05 for the comparison between TXA and control group

^§^*P* < 0.05 for the comparison between TXA and EACA group

### Comparison of TXA and EACA groups

THA patients who received EACA had greater intraoperative blood loss (*P* < 0.001), postoperative drainage (*P* = 0.011) and total blood loss (*P* < 0.001) compared with patients who received TXA (Fig. 1.). As such, significant difference was noted in the mean RBC units transfused per patient between the TXA and EACA groups, 0.21 units vs. 0.62 units, respectively (*P* < 0.05). Likewise, the TXA group required a smaller average number of millilitres transfused per patient and exhibited lower transfusion rates (10.6% vs. 20.6%, *P* < 0.05) (Table 2) when compared with EACA group. Moreover, change in HGB levels was appreciably bigger in EACA group than in TXA group (*P* = 0.016), while no significant difference was noted in changes in HCT levels per patient between the TXA and EACA groups (*P* = 0.112) (Fig. 2.).

### Safety outcomes

During the hospital stay, no thrombotic episodes for any hip replacement patient were seen. There were also no relevant complications, including deep venous thrombosis (DVT) and pulmonary embolism, that were observed during the 1-month follow-up. Thus, the 1-month readmission rate caused by relevant complications was 0. All these findings indicated that both antifibrinolytics are clinically safe.

### Cost comparison

In this study, all patients in the TXA and EACA groups received 2 g of TXA and 8 g of EACA, respectively. At our hospital, the costs for these TXA and EACA dosages were 102.76 RMB and 235.72 RMB, respectively. The cost for 1 unit of blood is 800 RMB in our country. The cost savings for transfusions when TXA is used would be 800 RMB/unit × reduction in average transfusion (1.13 – 0.21 units) = 736.00 RMB; EACA use would be: 800 RMB/unit × reduction in average transfusion (1.13 – 0.62 units) = 408.00 RMB. Thus, if the results from this study were generalized, the cost savings would be as follows: TXA, 736.00 RMB – 102.76 RMB = 633.24 RMB per patient; EACA, 408.00 RMB – 235.72 RMB = 172.28 RMB per patient (Table 2).

## Discussion

Currently, EACA was regarded as the preferred choice in orthopaedic surgeries for its lower cost per surgery and comparable transfusion rates with TXA in many medical institutions including Chinese hospitals, but the direct and indirect costs of both antifibrinolytics can vary from institution to institution. On the other hand, in a summary of the previous antifibrinolytics studies in THA in China (Supplementary table 1), we can find that there were only clinical experiments regarding TXA with no research on EACA, let alone the comparative study between TXA and EACA. So, no evidence demonstrated the cost advantage of EACA over TXA in Chinese areas to date. Therefore, the current study was performed to compare both medicines in a retrospective clinical study in a Chinese population of patients undergoing THA and to find the most economical and efficient antifibrinolytics adapted to China. The most important finding of this study is that TXA was demonstrated to be superior to EACA in perioperative blood management and medical cost in THA based on a retrospective study of 295 Chinese patients.

Previously, although abundant studies estimating perioperative blood loss and transfusion outcomes for patients with orthopaedic surgeries randomized to either TXA or EACA have been performed to date (Supplementary table 2), there was only one prospective, randomized controlled trial that has compared TXA and EACA for patients undergoing THA. Bradley et al. [12] reported results from 90 THA patients who were given TXA or EACA. No statistically significant differences were observed in any haematological outcome measure when using TXA or EACA. Likewise, others obtained similar results. However, our data with THA patients disagreed with these studies. Our results support the opinion that blood losses and transfusion rates are appreciably higher in the EACA group than in the TXA group and this difference was statistically remarkable. The reasons behind the divergence between our report and other literatures on the hemostatic effect of TXA and EACA can be concluded into the following two points. The first is the dose-ratio of both drugs. From Supplementary table 2, we may find apart from Morales-Avalos’ report, the maximum dose-ratio of TXA and EACA among other literatures is 1:5 which is smaller than that of 1:4 in our procedures. It would have meant there was a relative higher dosage of TXA and lower dosage of EACA in our study compared with other literatures. The second is the method of administration. Although dose-ratio of TXA and EACA in Morales-Avalos’ report is 1:1.5 which is greater than our usage, both drugs were administrated orally. The bioavailability of TXA and EACA after oral administration in humans is respectively 30 to 50% and 80% of the ingested dose, which means oral administration will greatly diminish the effect of TXA. Thus, relative higher dosage or preponderant method of administration of EACA may be the major cause of similar effect as TXA in perioperative blood management reported by precious literatures.

Currently, EACA was reported to be at a lower drug cost in many institutions than TXA [8]. Therefore, some studies have indicated that EACA seems to have higher clinical application value than TXA. However, direct drug cost was the only indicator considered when comparing medical costs, yet important indirect costs were neglected, such as transfusion-related costs. In addition, the medical cost between institutions also varies significantly. In a summary of the medication acquisition cost for TXA and EACA in American areas (Supplementary table 3), we can find that average cost of TXA per 2 g is obviously more expensive than that of EACA per 8 g. Unlike these data, the cost of both medicines is radically different in China. The medication cost for TXA and EACA from fourteen Chinese provinces were collected, which was displayed in the Supplementary table 4. From this table, it was found that average drug cost for EACA per 8 g is remarkedly higher than that for TXA per 2 g (228.86 RMB v.s. 104.70 RMB per surgery). While the difference in cost, approximately 124.16 RMB reported by us, may be unobvious on a per surgery basis, considering the increase of THA and potential sustained predominance of this procedure in China, the cumulative savings may be crucial for total Chinese healthcare system. Thus, it is necessary to select the optimum antifibrinolytics applicable to Chinese areas based on cost and regional availability.

The TXA preparation costs in our institution are approximately 102.76 RMB per 2 g, the EACA preparation costs are 235.72 RMB per 8 g, which is similar with other Chinese areas and may be regarded as the representative of Chinese areas. Considering similar safety profiles and stronger efficacy, the application of TXA can save up to 133 RMB per patient instead of EACA. Moreover, the results of the present study further confirmed that the use of TXA and EACA saved 633.24 RMB and 172.28 RMB per patient, respectively, based on drug and transfusion costs. The difference in cost savings for both drugs was statistically significant. Therefore, when comprehensively considering drug and transfusion costs, the application of TXA will cause cost savings of 460.96 per patient undergoing THA compared with EACA. Thus, all these findings indicated TXA to be more economical and effective than EACA in THA in our hospital.

On the other hand, surgery time is also involved in the cost of the surgeries. However, whether antifibrinolytics can shorten operative time has not been clearly demonstrated. Significantly, our study originally found EACA (74.80 $\pm$ 29.795 min v.s. 89.45 $\pm$ 39.975 min, *P* < 0.05) or TXA (70.00 $\pm$ 17.629 min v.s. 89.45 $\pm$ 39.975 min, *P* < 0.05) can cause the significantly reduced operative time when compared with control group, which showed the superiority of antifibrinolytics again in THA.

Antifibrinolytics have not been demonstrated to be linked to an increase in the rate of DVT [13]. Similarly, no relevant complications, including DVT and pulmonary embolism, were observed in any study patients, indicating that the treatment is safe when applied using our approach. One of the limitations in the current study is that we did not perform echo-Doppler systematically on all patients enrolled in the trial for the diagnosis of DVT. Nevertheless, no clinically relevant thromboembolic events were found during the 1-month follow-up period. In addition, no significant difference in the length of hospital stay among the three groups was noted, indicating that antifibrinolytics did not have an impact on the hospital stays.

Some potential limitations of the current study should be noticed. As a retrospective study with small sample sizes at a single institution, the results can be influenced by plenty of factors when extended to the whole country. Besides, causality cannot be demonstrated by this study due to the possibility of bias. Importantly, the inclusion of patients with a femoral neck fracture is probably debatable for the interpretation of results. To help manage this, a statistical analysis was further performed to evaluate the effect of antifibrinolytics in non-fracture patients undergone THA. The results demonstrated EACA and TXA can significantly reduce perioperative blood loss and transfusion rates (Supplementary Figs. 1 and 2). Thus, controversy has not been caused by the inclusion of patients with fracture. Lastly, the results of cost analysis from a single healthcare system’s cost may lack external applicability and validity because of the difference of product volume and negotiated contracts among distinct healthcare system. Hence, a prospective, double-blind, multicentre trials with large sample sizes in China was required to further confirm our idea in the coming years.

## Conclusions

The application of perioperative antifibrinolytics notably reduces the need for perioperative blood transfusions. What’s more, this study demonstrated that TXA (2 g) is superior to EACA (8 g) for decreasing blood loss, transfusions, as well as costs. These results indicate that TXA may be the optimum antifibrinolytics for THA than EACA.

## Supplementary Information

**Additional file 1.** Supplemental tables.

**Additional file 2.** Supplementary figures.

## Data Availability

The datasets generated and analysed during the current study are not publicly available due recognisable patient information but are available from the corresponding author on reasonable request with ease.

## Abbreviations

DVT: Deep venous thrombosis
EACA: Epsilon aminocaproic acid
HCT: Haematocrit
HGB: Haemoglobin
RBC: Red blood cell
THA: Total hip arthroplasty
TXA: Tranexamic acid
