# Supplementary material for: Tranexamic versus aminocaproic acids in patients with total hip arthroplasty: a retrospective study
Source: BMC Musculoskelet Disord. 2022 Nov 19;23:999. doi: 10.1186/s12891-022-05922-5 (PMC9675136; doi:10.1186/s12891-022-05922-5)
Supplement: Supplementary file 1 — Additional file 1. Supplemental tables. [file 12891_2022_5922_MOESM1_ESM.pdf]

Supplementary table 1 Overview of Relevant Literature Regarding Tranexamic Acid in THA in China

Authors (y)	Location	Study Type	Objective	Conclusion
Wei et al (2014)[1]	Guangzhou	RCT	To compare the hemostatic effect of topical and intravenous TXA in THA.	Topical use of TXA was equally effective and safe compared with intravenous TXA.
Yue et al (2014)[2]	Chengdu	RCT	To assess the hemostatic effect of a high-dose 3 g topical TXA in THA.	3 g topical TXA was effective and safe in reducing bleeding and transfusions.
Chang et al (2014)[3]	Taiwan	RCS	To assess the hemostatic effect of topical TXA in THA.	Topical TXA reduces postoperative bleeding and decreases blood transfusion rates.
Wang et al (2016)[4]	Chengdu	RCT	To evaluate the hemostatic effects of different doses of intravenous TXA.	15mg/kg TXA was more effective than 10 mg/kg TXA in perioperative blood management.
Wu et al (2016)[5]	Chengdu	RCT	To evaluate whether the combination of erythropoietin and TXA is superior to TXA alone.	Erythropoietin combined with TXA is superior to TXA alone in perioperative blood management.
Xie et al (2016)[6]	Chengdu	RCT	To compare the efficacy and safety of the combined use of TXA with the intravenous or local use alone.	Combined TXA regimen is more effective in decreasing blood loss than intravenous or local administration alone.
Yi et al (2016)[7]	Chengdu	RCT	To assess the efficacy and safety of intravenous administration combined with topical administration of TXA.	Intravenous combined with topical administration of TXA significantly reduced postoperative bleeding and the transfusion rate.
Zhang et al (2016)[8]	Luoyang	RCT	To investigate the hemostatic impact of topical injection of TXA as well as short-term safety and adverse effects compared with intravenous administration of TXA.	Preoperative intravenous TXA and postoperative topical TXA significantly reduced postoperative blood loss and transfusion rates and the short-term safety was good.
Wu et al (2016)[9]	Chengdu	RCT	To evaluate the efficacy and safety of combination of intravenous and topical TXA in revision THA.	Combined administration of intravenous and topical TXA can effectively decrease total blood loss and number of blood transfusions required compared with intravenous TXA alone.
Xie et al (2017)[10]	Chengdu	RCT	To examine the efficacy and safety of multiple boluses of intravenous TXA on the hidden blood loss and inflammatory response.	A regime of three boluses leads to a smaller decrease in the level of Hb, less post-operative inflammation and a shorter length of stay in hospital than a single bolus.

Luo et al (2018)[11]	Chengdu	RCT	To compare the hemostatic effect of topical, intravenous and topical TXA in THA.	Oral TXA is recommended because of its cost-benefit superiority and ease of administration
Cao et al (2018)[12]	Chengdu	RCT	To determine whether the administration of multiple boluses of oral and intravenous TXA postoperatively was equivalent in reducing blood loss.	Multiple boluses of oral TXA and intravenous TXA postoperatively are equivalent in reducing blood loss, Hb and Hct drop.
Wang et al (2019)[13]	Chengdu	RCT	To identify the most effective regimen of multiple-dose oral TXA in achieving maximum reduction of blood loss and inflammatory response.	The postoperative four-dose regimen brings about maximum efficacy in reducing blood loss, alleviating inflammatory response and improving analgesia and immediate recovery.
Huang et al (2019)[14]	Chengdu	RCS	To analyze clinical outcomes and complications rate after total joint arthroplasty of the lower limbs using TXA or not.	The hemophiliacs treated with TXA had less perioperative blood loss, hidden blood loss, transfusion rate, a lower ratio of postoperative knee swelling, etc.
Wu et al (2020)[15]	Chongqing	RCS	To compare the efficacy of a three-day prolonged course of multiple-dose of TXA with a single pre-operative dose of TXA.	A three-day prolonged-course of multiple dose of TXA was consistently effective in reducing postoperative Hb drops, estimated total blood loss, inflammatory responses.

RCT, randomized controlled trial; RCS, retrospective comparative study; THA, total hip arthroplasty; TXA, tranexamic acid; Hb, hemoglobin; Hct, hematocrit.

Supplementary table 2 Overview of Relevant Literature Regarding the Contrast Research of TXA and EACA in Orthopedic Surgery

**Authors**	**Location**	**Type**	**Surgery**	**Inventions**					**Outcomes**
				**Control**	**TXA**	**EACA**	**Route**	**Dose-ratio of TXA and EACA**	
Camarasa et al (2006)[16]	Spain	RCT	TKA	NS	10 mg/kg	100 mg/kg	i.v.	1:10	Antifibrinolytic agents produce a significant decrease in blood loss in patients undergoing total knee replacement, reflected in a reduction in the number of blood transfusions required.
Verma et al (2014)[17]	USA	RCT	SS	NS	10 mg/kg	100 mg/kg	i.v.	1:10	TXA and EACA reduced operative blood loss but not transfusion rate. TXA is more effective at reducing postoperative drainage and total blood losses compared with EACA.
Peters et al (2015)[18]	USA	RCT	SS	NS	10 mg/kg	100 mg/kg	i.v.	1:10	The findings in this study support the use of antifibrinolytics to reduce blood loss in posterior adult spinal deformity surgery.
Churchill et al (2016)[19]	USA	RCS	THA	NS	10 mg/kg	100/200 mg/kg	i.v.	1:10/20	Intraoperative antifibrinolytic use significantly decreases need for postoperative blood transfusions. At our institution, EACA is comparable to TXA in THA for reducing transfusion rates while at a lower cost per surgery.
Boese et al (2017)[20]	USA	RCT	TKA	NS	1g in 250 mL NS	7g in 250 ml NS	i.v.	1:7	Although the estimated blood loss was significantly greater in the EACA group, no transfusions were required and no significant between-group differences were observed for any other outcomes measured. We concluded that EACA may be an acceptable alternative to TXA for blood conservation

									following TKA, although replication of our results in noninferiority trials is necessary.
Bradley et al (2019)[21]	USA	RCT	TJA	NS	1 g in 250 ml NS	5 g in 250 ml NS	i.v.	1:5	For hip and knee arthroplasty procedures, EACA is associated with increased perioperative blood loss compared with TXA. However, there is no significant difference in transfusion rate.
Morales-Avalos et al (2021)[22]	México	RCT	TKA	No	1,300 mg	2,000 mg	oral	1:1.5	Although patients who received TXA presented an average of 140 mL less blood loss than patients in the EACA group, the difference did not appear to be clinically important, the transfusion rate was very low, and there were no between-group differences in postoperative complications.

RCT, randomized controlled trial; RCS, retrospective comparative study; THA, total hip arthroplasty; TKA, total knee arthroplasty; SS, spinal surgery; TJA, total joint arthroplasty; TXA, tranexamic acid; EACA, aminocaproic acid; Hb, hemoglobin; Hct, hematocrit; i.v., intravenous injection;

Supplementary table 3 The medication acquisition cost for Tranexamic and Aminocaproic acids in American areas

	Location	Cost (USD) per 2 g		Province	Cost (USD) per 8 g
	New York[23]	40-50		New York[23]	1.6-3.2
	Milan[24]	2		Milan[24]	2.40
**Tranexamic acid**	Massachusetts[25]	25.88	**Aminocaproic acid**	Cleveland[26]	4.48-8.96
	Rhode Island[27]	116		California[19]	1.648
	Wisconsin[19]	79.16		Wisconsin[19]	21.6
	Maryland[28]	24.22			

Supplemental table 4 The medication acquisition cost for Tranexamic and Aminocaproic acids in Chinese areas

	Province	Cost (RMB) per 2 g		Province	Cost (RMB) per 8 g
**Tranexamic acid**	Jilin	105.96	**Aminocaproic acid**	Szechwan	256.00
	Chungking	105.24		Jiangxi	292.00
	Zhejiang	102.56		Liaoning	235.72
	Shanxi	102.56		Shanxi	182.00
	Kweichow	105.90		Kweichow	182.00
	Kansu	106.10		Kansu	182.00
	Hainan	108.86		Hainan	292.00
	Jiangsu	102.56		Guangxi	256.00
	Beijing	102.56		Inner Mongolia	182.00
	**Average**	**104.70**		**Average**	**228.86**

Reference

1. Wei W, Wei B: Comparison of topical and intravenous tranexamic acid on blood loss and transfusion rates in total hip arthroplasty. *J Arthroplasty.* 2014; **29**(11):2113-2116.

2. Yue C, Kang P, Yang P, Xie J, Pei F: Topical application of tranexamic acid in primary total hip arthroplasty: a randomized double-blind controlled trial. *J Arthroplasty.* 2014; **29**(12):2452-2456.

3. Chang CH, Chang Y, Chen DW, Ueng SW, Lee MS: Topical tranexamic acid reduces blood loss and transfusion rates associated with primary total hip arthroplasty. *Clin Orthop Relat Res.* 2014; **472**(5):1552-1557.

4. Wang C, Kang P, Ma J, Yue C, Xie J, Pei F: Single-dose tranexamic acid for reducing bleeding and transfusions in total hip arthroplasty: A double-blind, randomized controlled trial of different doses. *Thromb Res.* 2016; **141**:119-123.

5. Wu YG, Zeng Y, Shen B, Si HB, Cao F, Yang TM, Pei FX: Combination of erythropoietin and tranexamic acid in bilateral simultaneous total hip arthroplasty: a randomised, controlled trial. *Hip Int.* 2016; **26**(4):331-337.

6. Xie J, Ma J, Yue C, Kang P, Pei F: Combined use of intravenous and topical tranexamic acid following cementless total hip arthroplasty: a randomised clinical trial. *Hip Int.* 2016; **26**(1):36-42.

7. Yi Z, Bin S, Jing Y, Zongke Z, Pengde K, Fuxing P: Tranexamic Acid Administration in Primary Total Hip Arthroplasty: A Randomized Controlled Trial of Intravenous Combined with Topical Versus Single-Dose Intravenous Administration. *J Bone Joint Surg Am.* 2016; **98**(12):983-991.

8. Zhang Y, Zhang L, Ma X, Jia Y, Wang H, Zhu Y, Liu Y: What is the optimal approach for tranexamic acid application in patients with unilateral total hip arthroplasty? *Orthopade.* 2016; **45**(7):616-621.

9. Wu YG, Zeng Y, Yang TM, Si HB, Cao F, Shen B: The Efficacy and Safety of Combination of Intravenous and Topical Tranexamic Acid in Revision Hip Arthroplasty: A Randomized, Controlled Trial. *J Arthroplasty.* 2016; **31**(11):2548-2553.

10. Xie J, Hu Q, Ma J, Huang Q, Pei F: Multiple boluses of intravenous tranexamic acid to reduce hidden blood loss and the inflammatory response following enhanced-recovery primary total hip arthroplasty: a randomised clinical trial. *Bone Joint J.* 2017; **99-B**(11):1442-1449.

11. Luo ZY, Wang HY, Wang D, Zhou K, Pei FX, Zhou ZK: Oral vs Intravenous vs Topical Tranexamic Acid in Primary Hip Arthroplasty: A Prospective, Randomized, Double-Blind, Controlled Study. *J Arthroplasty.* 2018; **33**(3):786-793.

12. Cao G, Huang Z, Xie J, Huang Q, Xu B, Zhang S, Pei F: The effect of oral versus intravenous tranexamic acid in reducing blood loss after primary total hip arthroplasty: A randomized clinical trial. *Thromb Res.* 2018; **164**:48-53.

13. Wang D, Yang Y, He C, Luo ZY, Pei FX, Li Q, Zhou ZK, Zeng WN: Effect of Multiple Doses of Oral Tranexamic Acid on Haemostasis and Inflammatory Reaction in Total Hip Arthroplasty: A Randomized Controlled Trial. *Thromb Haemost.* 2019; **119**(1):92-103.

14. Huang ZY, Huang Q, Zeng HJ, Ma J, Shen B, Zhou ZK, Pei FX: Tranexamic acid may benefit patients undergoing total hip/knee arthroplasty because of haemophilia. *BMC Musculoskelet Disord.* 2019; **20**(1):402.
15. Wu XD, Tian M, He Y, Chen Y, Tao YZ, Shao L, Luo C, Xiao PC, Zhu ZL, Liu JC *et al*: Efficacy of a three-day prolonged-course of multiple-dose versus a single-dose of tranexamic acid in total hip and knee arthroplasty. *Ann Transl Med.* 2020; **8**(6):307.
16. Camarasa MA, Ollé G, Serra-Prat M, Martín A, Sánchez M, Ricós P, Pérez A, Opisso L: Efficacy of aminocaproic, tranexamic acids in the control of bleeding during total knee replacement: a randomized clinical trial. *Br J Anaesth.* 2006; **96**(5):576-582.
17. Verma K, Errico T, Diefenbach C, Hoelscher C, Peters A, Dryer J, Huncke T, Boenigk K, Lonner BS: The relative efficacy of antifibrinolytics in adolescent idiopathic scoliosis: a prospective randomized trial. *J Bone Joint Surg Am.* 2014; **96**(10):e80.
18. Peters A, Verma K, Slobodyanyuk K, Cheriyan T, Hoelscher C, Schwab F, Lonner B, Huncke T, Lafage V, Errico T: Antifibrinolytics reduce blood loss in adult spinal deformity surgery: a prospective, randomized controlled trial. *Spine (Phila Pa 1976).* 2015; **40**(8):E443-449.
19. Churchill JL, Puca KE, Meyer ES, Carleton MC, Truchan SL, Anderson MJ: Comparison of ε-Aminocaproic Acid and Tranexamic Acid in Reducing Postoperative Transfusions in Total Hip Arthroplasty. *J Arthroplasty.* 2016; **31**(12):2795-2799.
20. Boese CK, Centeno L, Walters RW: Blood Conservation Using Tranexamic Acid Is Not Superior to Epsilon-Aminocaproic Acid After Total Knee Arthroplasty. *J Bone Joint Surg Am.* 2017; **99**(19):1621-1628.
21. Bradley KE, Ryan SP, Penrose CT, Grant SA, Wellman SS, Attarian DE, Green CL, Risoli T, Jr., Bolognesi MP: Tranexamic acid or epsilon-aminocaproic acid in total joint arthroplasty? A randomized controlled trial. *Bone Joint J.* 2019; **101-B**(9):1093-1099.
22. Morales-Avalos R, Ramos-Morales T, Espinoza-Galindo AM, Garay-Mendoza D, Peña-Martínez VM, Marfil-Rivera LJ, Garza-Ocañas L, Acosta-Olivo C, Cerda-Barbosa JK, Valdés-González NL *et al*: First Comparative Study of the Effectiveness of the Use of Tranexamic Acid against ε-Aminocapróic Acid via the Oral Route for the Reduction of Postoperative Bleeding in TKA: A Clinical Trial. *J Knee Surg.* 2021; **34**(4):383-405.
23. Eaton MP: Antifibrinolytic therapy in surgery for congenital heart disease. *Anesth Analg.* 2008; **106**(4):1087-1100.
24. Casati V, Guzzon D, Oppizzi M, Cossolini M, Torri G, Calori G, Alfieri O: Hemostatic effects of aprotinin, tranexamic acid and epsilon-aminocaproic acid in primary cardiac surgery. *Ann Thorac Surg.* 1999; **68**(6):2252-2256; discussion 2256-2257.
25. Reid RW, Zimmerman AA, Laussen PC, Mayer JE, Gorlin JB, Burrows FA: The efficacy of tranexamic acid versus placebo in decreasing blood loss in pediatric patients undergoing repeat cardiac surgery. *Anesth Analg.* 1997; **84**(5):990-996.
26. Eubanks JD: Antifibrinolytics in major orthopaedic surgery. *J Am Acad Orthop Surg.* 2010; **18**(3):132-138.
27. Tuttle JR, Ritterman SA, Cassidy DB, Anazonwu WA, Froehlich JA, Rubin LE: Cost benefit analysis of topical tranexamic acid in primary total hip and knee arthroplasty. *J*

Arthroplasty. 2014; **29**(8):1512-1515.
28. Ehresman J, Pennington Z, Schilling A, Medikonda R, Huq S, Merkel KR, Ahmed AK, Cottrill E, Lubelski D, Westbroek EM *et al*: Cost-benefit analysis of tranexamic acid and blood transfusion in elective lumbar spine surgery for degenerative pathologies. *J Neurosurg Spine.* 2020:1-9.
