# Supplementary material for: Tranexamic versus aminocaproic acids in patients with total hip arthroplasty: a retrospective study
Source: BMC Musculoskelet Disord. 2022 Nov 19;23:999. doi: 10.1186/s12891-022-05922-5 (PMC9675136; doi:10.1186/s12891-022-05922-5)
Supplement: Supplementary file 2 — Additional file 2. Supplementary figures. [file 12891_2022_5922_MOESM2_ESM.docx]

**Supplementary figures**

This statistical analysis was performed to evaluate the effect of antifibrinolytics in non-fracture patients undergone THA (Control group, n=82; EACA, n=68; TAX, n=63). The results demonstrated EACA and TXA can significantly reduce perioperative blood loss and transfusion rates (Supplementary Figure 1 and 2).


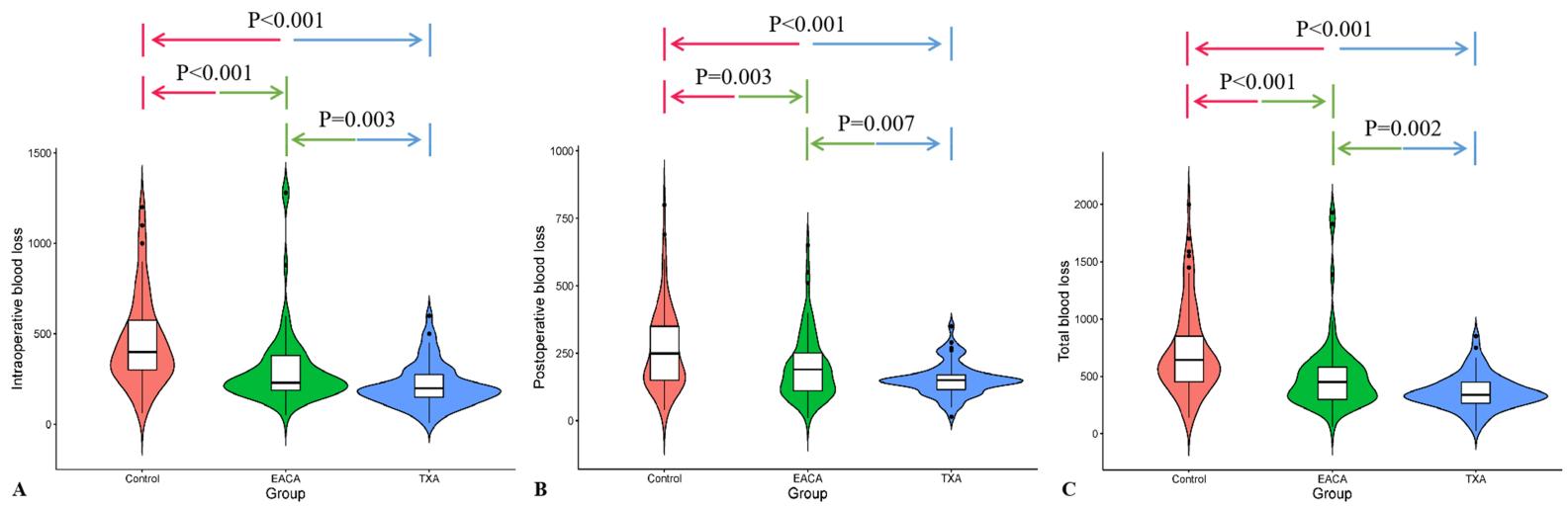


**Supplementary Figure 1. Comparison of perioperative blood loss in non-fracture patients among three groups.**

(A) Intraoperative blood loss. (B) Postoperative blood loss. (C) Total blood loss. Statistical significance was reached for total losses among tranexamic acid (TXA), epsilon aminocaproic acid (EACA), and placebo. Pairwise comparisons among the three groups were determined by Tukey's test.


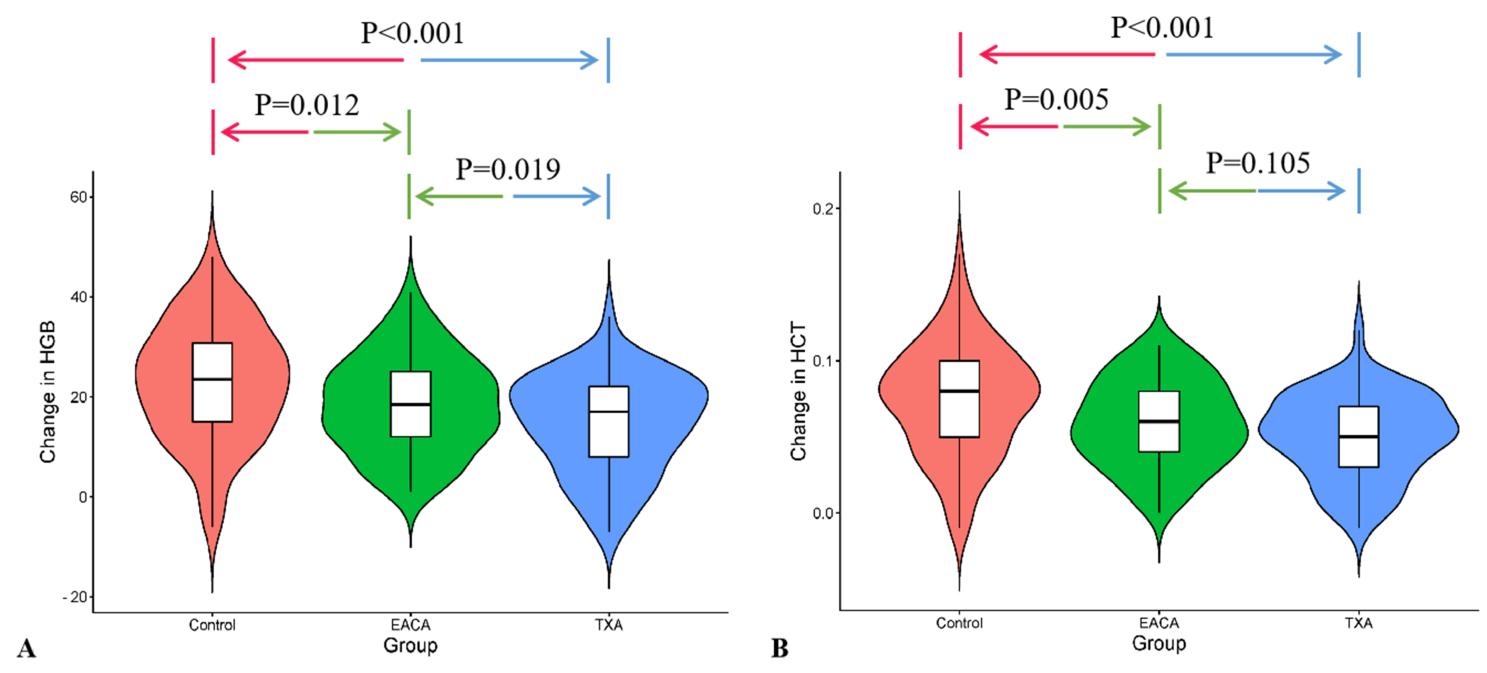


**Supplementary Figure 2. Change in haemoglobin (HGB) and haematocrit (HCT) levels in non-fracture patients among the three groups.**

(A) Change of HGB level; (B) Change of HCT level. TXA, tranexamic acid; EACA, epsilon aminocaproic acid. Pairwise comparisons among the three groups were determined by Tukey's test.
